# Genome Sequence of *Saccharomyces carlsbergensis*, the World’s First Pure Culture Lager Yeast

**DOI:** 10.1534/g3.113.010090

**Published:** 2014-02-27

**Authors:** Andrea Walther, Ana Hesselbart, Jürgen Wendland

**Affiliations:** Carlsberg Laboratory, Yeast Biology, Gamle Carlsberg Vej 10, Valby, Denmark

**Keywords:** genome sequencing, genome evolution, chromosome loss, ploidy, fermentation

## Abstract

Lager yeast beer production was revolutionized by the introduction of pure culture strains. The first established lager yeast strain is known as the bottom fermenting *Saccharomyces carlsbergensis*, which was originally termed *Unterhefe* No. 1 by Emil Chr. Hansen and has been used in production in since 1883. *S. carlsbergensis* belongs to group I/Saaz-type lager yeast strains and is better adapted to cold growth conditions than group II/Frohberg-type lager yeasts, *e.g.*, the Weihenstephan strain WS34/70. Here, we sequenced *S. carlsbergensis* using next generation sequencing technologies. Lager yeasts are descendants from hybrids formed between a *S. cerevisiae* parent and a parent similar to *S. eubayanus*. Accordingly, the *S. carlsbergensis* 19.5-Mb genome is substantially larger than the 12-Mb *S. cerevisiae* genome. Based on the sequence scaffolds, synteny to the *S. cerevisae* genome, and by using directed polymerase chain reaction for gap closure, we generated a chromosomal map of *S. carlsbergensis* consisting of 29 unique chromosomes. We present evidence for genome and chromosome evolution within *S. carlsbergensis* via chromosome loss and loss of heterozygosity specifically of parts derived from the *S. cerevisiae* parent. Based on our sequence data and via fluorescence-activated cell-sorting analysis, we determined the ploidy of *S. carlsbergensis*. This inferred that this strain is basically triploid with a diploid *S. eubayanus* and haploid *S. cerevisiae* genome content. In contrast the Weihenstephan strain, which we resequenced, is essentially tetraploid composed of two diploid *S. cerevisiae* and *S. eubayanus* genomes. Based on conserved translocations between the parental genomes in *S. carlsbergensis* and the Weihenstephan strain we propose a joint evolutionary ancestry for lager yeast strains.

Starting from the early ages of agriculture and the domestication of barley, fermented beverages played an important role in the emerging societies. Beer has been known for millennia dating back at least to the Sumerians 6000 BC. Fermented beverages provided not only nutrition but were basically the only sources of uncontaminated clean liquids and thus of medicinal value. Although there is a plethora of microorganisms within the *Saccharomyces* complex that can be found in natural fermentations, *Saccharomyces cerevisiae* has been the predominant species in certain types of fermentations, *e.g.*, in ale beers and in wine.

Today, however, most beer volume is generated with lager beers. Lager brewing was initiated in Bavaria in the 15th century (Libkind *et al.* 2011). The German Reinheitsgebot from 1516 regulated that beer should only be made of water, malt, and hops without any other ingredients—of course at that time *S. cerevisiae* was not known. Yet, lager beer production differed markedly from ale brewing by its substantially lower fermentation temperatures—starting as low as 5°. In the 19th century, lager beer gained so much popularity that keeping up production required a break with tradition. Supported by the invention of refrigeration, lager beer was then also produced in the summer months, which traditionally had been considered the off-season.

However, beer spoilage of lager beers became increasingly frequent over summer due to contamination with wild yeasts. This led to the scientific investigation of this problem by Louis Pasteur and Emil Chr. Hansen. Hansen verified that wort became infected by wild yeasts and therefore devised a method to isolate pure cultures of yeast strains (Hansen 1883). One of these strains, *Unterhefe* No. 1, showed a very convincing brewing performance and was thus chosen as production strain at the Carlsberg brewery in 1883 and given freely to other breweries by its owner J. C. Jacobsen and later entered the CBS strain collection in 1947.

Lager yeasts are interspecies hybrids between *S. cerevisiae* and *S. uvarum* parents (Nilsson-Tillgren *et al.* 1986; Kielland-Brandt *et al.* 1995; Casaregola *et al.* 2001; Bond 2009). The first lager yeast draft genome sequence was that of the Weihenstephan (WS34/70) strain, demonstrating the allotetraploid hybrid nature of this lager yeast (Nakao *et al.* 2009). Previous analyses of lager yeast strains indicated that different isolates contain different gene or chromosome sets (Hansen and Kielland-Brandt 1994; Fujii *et al.* 1996; Borsting *et al.* 1997; Tamai *et al.* 1998; Yamagishi and Ogata 1999). Using polymerase chain reaction (PCR)-restriction fragment length polymorphism, two types of lager yeasts could be distinguished. On the one hand there were lager strains currently used in production that showed almost a complete set of both of the parental genomes, and on the other a set of lager yeast strains, including *S. carlsbergensis* and *S. monacensis*, that were found to lack certain portions of the *S. cerevisiae* genome (Rainieri *et al.* 2006). By means of array-based comparative genomic hybridization (array-CGH), this partition into two groups was further refined. This indicated that regional distribution matches the gene content and suggested that group I corresponds to the Saaz type, whereas group 2 is represented by the Frohberg type. It was also suggested that two independent hybridization events generated the two types of lager yeast (Dunn and Sherlock 2008).

The origin of the non-*cerevisiae* parent in lager yeast has long been debated. Recently, the isolation of *S. eubayanus* from southern beech (*Nothofagus*) of Pathagonian forests provided one potential resource of a strain that upon hybridization with *e.g.*, an *S. cerevisiae* ale yeast could have generated lager yeast hybrids (Dunn and Sherlock 2008; Libkind *et al.* 2011). Throughout this paper, we refer to the non-*cerevisiae* part of lager yeast genomes as *S. eubayanus*, instead of *S. uvarum* or *S. eubayanus*-like, etc.

Here we report the genome sequence and analysis of the first pure culture lager yeast production strain *S. carlsbergensis* and a genome scale comparison of this strain with the Weihenstephan yeast WS34/70.

## Materials and Methods

### Strains, media, and fermentation setup

The following strains were used in this study: *Saccharomyces carlsbergensis*, CBS 1513; *Saccharomyces monacensis*, CBS 1503; *Saccharomyces eubayanus*, CBS 12357; *Saccharomyces cerevisiae* CEN.PK; *Saccharomyces pastorianus*, Weihenstephan WS34/70; and *Saccharomyces cerevisiae* ale yeast (Carlsberg collection). Growth assays were performed in Yeast Extract Peptone Dextrose medium (1% yeast extract, 2% peptone, 2% glucose) at various temperatures. Strains were inoculated with an initial OD_600_ (*i.e.*, the optical density of a sample measured at a wavelength of 600 nm) of 0.1 and then grown for 2−4 d shaking. Industrial brewing conditions are produced by small-scale fermentations in tall tube cylinders with 200 mL of volume. 14° Plato granmalt (150 g/L malt granules, 5 g/L yeast extract) was fermented with selected strains at 14°. Yeast strains were propagated in granmalt prior to pitching with an OD_600_ of 0.2. Stirring of the fermentation cyclinders was set to 190 rpm. The fermentation performance was followed by online measuring of CO_2_ loss and wort density using an Anton Paar DMA 35 densitometer measuring gravity (*i.e.*, amount of sugars) in °P (refers to the percentage of sucrose by weight). The end of fermentation was reached when the sugar concentration did not decrease further for 2 d. All fermentations were conducted in biological triplicates. At the end of fermentation, the alcohol concentration was measured using an Alcolyzer M (Alcolyzer Beer Analyzing System; Anton Paar). A volume of 100 mL was used for flavor analysis.

### Flavor analysis

Samples of 100 mL were removed at the end of fermentation for analysis of aroma compounds. Alcohols and esters were measured by solvent extraction with carbon disulfide. After the samples were stirred for 30 min, they were centrifuged, and a volume of 2 µL of the lipid organic phase was directly injected into the gas chromatograph (GC; Agilent 6890). 1-Octanol served as an internal standard. Volatiles were separated on a DBWAX capillary column (30 m × 0.32 mm × 0.25 µm) and detected by a flame ionization detector (FID).

### Sequencing strategy and chromosomal assembly

Genome sequencing of *S. carlsbergensis* was performed using 454 GS FLX + sequencing of single reads and of a mate-pair library of 8-kb inserts. A fragment library and the additional 8-kb paired-end library were constructed with Rapid Library Prep Kit. An initial number of 635,399 reads and 480,966 paired end reads of an 8-kb library were assembled into 386 contigs and further combined into 78 scaffolds. Assembly into whole chromosomes was based on synteny to *S. cerevisiae* and *S. eubayanus* or directed PCR fragments were obtained to merge scaffolds. Primers are listed in Supporting Information, Table S1. The WS34/70 strain was resequenced using Illumina Miseq also including an 8-kb mate-pair library.

### Analysis of the *S. carlsbergensis* genome

The *S. carlsbergensis* Whole Genome Shotgun project has been deposited at DDBJ/EMBL/GenBank under the accession AZCJ00000000. The version described in this paper is version AZCJ01000000. The Weihenstephan WS34/70 Whole Genome Shotgun project has been deposited at DDBJ/EMBL/GenBank under the accession AZAA00000000. The version described in this paper is version AZAA01000000. For the visualization of chromosomal rearrangements in a Circos plot, a pairwise comparison of *S. carlsbergensis* and *S. cerevisiae* chromosomes was performed. The information about all chromosomal rearrangements was then synthesized in a tabular matrix which can be represented in a circular plot using the Circos software package (Krzywinski *et al.* 2009).

Ploidy analysis was visualized using a violin plot. As a first step the shotgun reads were aligned using the LASTZ program (http://www.bx.psu.edu/~rsharris/lastz/). Then its output was parsed with SAMtools (Li *et al.* 2009) to index and extract the number of aligned reads at each locus. A running average of mapped reads per window was calculated. The violin plot shows the distribution of the log2 ratios of copy number variation across each chromosome. The plot is generated using ggplot2 in R (Ito and Murphy 2013; R Development Core Team 2013). Scaffold alignments and sequencing analysis of PCR products were done using Lasergene DNAstar 11 (www.dnastar.com).

### Fluorescence-activated cell sorting (FACS) analysis

Ploidy analysis was confirmed using FACS. Cells were grown overnight (o/n) at room temperature to an end-exponential growth state. For the staining of cells with propidium iodine, cultures were first washed and resuspended in 1× SSC buffer before fixation in 70% ethanol at −20° o/n. Samples were then treated with RNAse o/n at 37° followed by proteinase K treatment at 50° for 1 hr. A final concentration of 3 µg/mL propidium iodine was added to each sample and incubated for 18 hr in darkness before FACS analysis (www.aragonlab.com/Protocols-Yeast.html).

## Results

### Growth and fermentation characteristics of lager yeast strains

**Figure 1:**
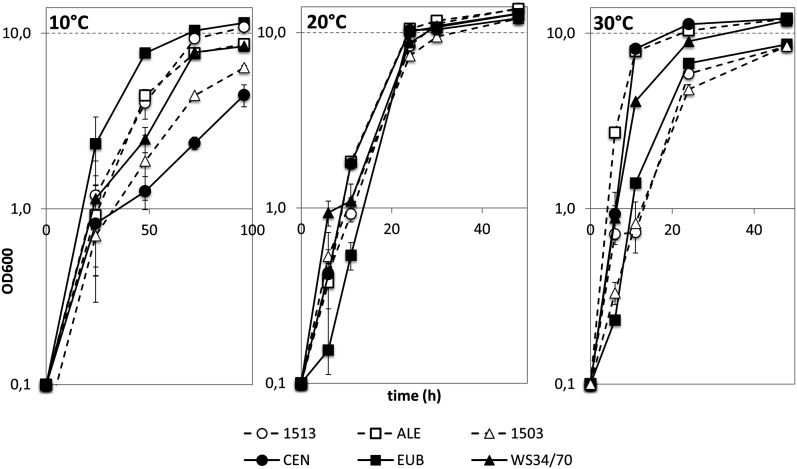
Growth comparisons with lager yeasts. Growth curves were obtained from YPD cultures grown at 10°, 20°, and 30° over a period of 2−4 d. The following strains were used: Group I lager yeast *S. carlsbergensis* (1513), *S. monacensis* (1503), group II lager yeast (WS34/70), an *S. cerevisiae* ale yeast (ALE), *S. eubayanus* (EUB), and the laboratory *S. cerevisiae* strain CEN.PK.

**Figure 2:**
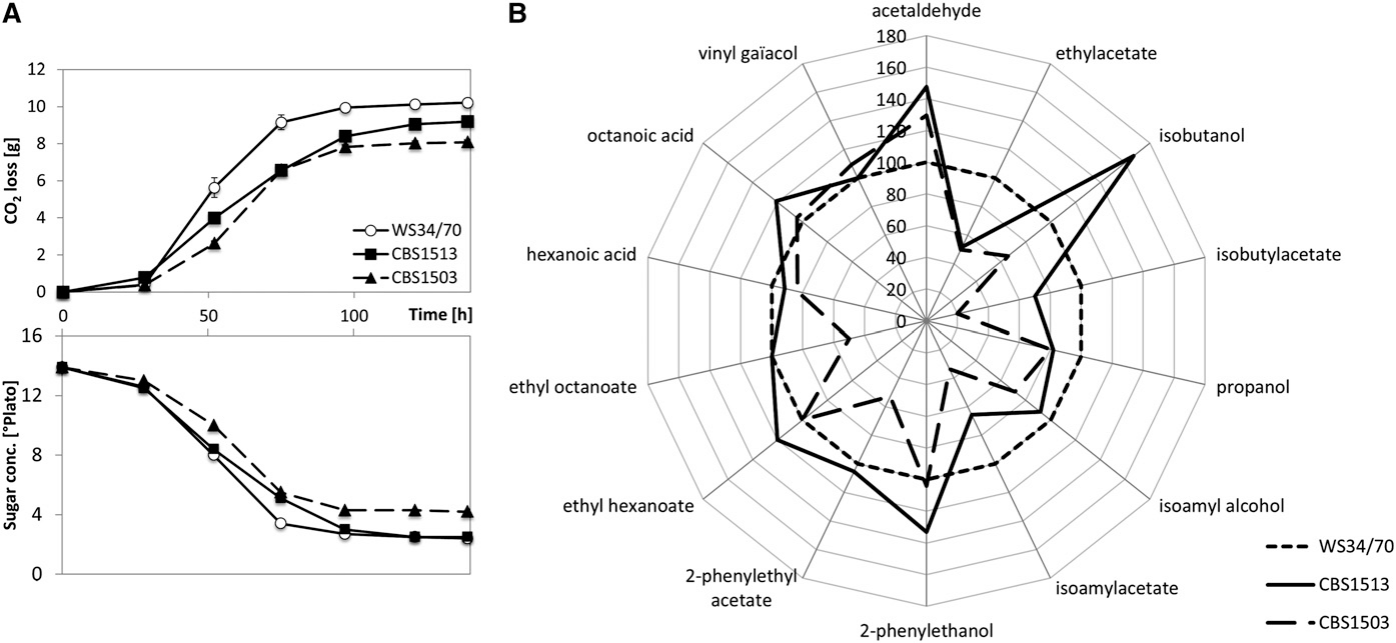
Malt-based fermentations and volatile compound analysis with lager yeast strains. (A) Representative fermentation kinetics of *S. carlsbergensis* (CBS1513), *S. monacensis* (CBS1503), and the Weihenstephan strain (WS34/70) with granulated malt of 14 °P at 14°. Data were averaged based on n > 4 parallel fermentations. The upper plot shows the weight loss over time based on CO_2_ release (g/L) the lower chart indicates the reduction of sugar content. (B) Spider chart representing the volatile flavors analyzed by GC/FID. The values obtained for the Weihenstephan strain were set to 100% (see Table S2 for details) and compared with those from the group I lager yeasts.

Lager yeasts are currently grouped into two categories, group I/Saaz and group II/Frohberg. We compared growth and fermentation characteristics of two group I strains, *S. carlsbergensis* and *S. monacensis* and the group II Weihenstephan WS34/70 strain with *S. eubayanus*, an ale yeast and the CEN.PK laboratory yeast strain. At low temperatures of 10°, *S. eubayanus* had a short lag phase and relatively rapid growth. This profile was best matched by *S. carlsbergensis*. At 20°, all assayed strains grouped closely together. At greater temperatures, however, *S. cerevisiae* and the ale yeast strain showed better growth compared with *S. eubayanus* and both group I lager yeasts. The Weihenstephan lager yeast showed intermediate growth rates at the upper and lower end of the temperature range (Figure 1). This finding indicates that *S. carlsbergensis* is better adapted to cold fermentation temperatures than the group II lager yeast/Weihenstephan strain. Historically, lager beer fermentation was carried out at very low temperatures (as low as 5°). Currently, however, greater fermentation temperatures are applied in industry. Here, we assayed fermentation performance at 14° using 14 °P wort. Under these conditions the group II lager yeast was fastest in fermentation and wort attenuation. Among the group I lager yeasts, *S. carlsbergensis* was faster than *S. monacensis* and reached the same attenuation level as the Weihenstephan strain (Figure 2A). These results indicate that group II lager yeasts are better adapted to greater temperature fermentation conditions than group I yeasts. Two main postfermentation parameters of industrial importance are the percentage of surviving cells and the ratio of petite cells among them. This is because for the setup of a second fermentation yeast cells from a previous fermentation are used as inoculum (termed “repitching”) and due to the inferior fermentation performance of petite cells. Survival rates of group I and II lager yeasts at the end of fermentation were similar. Yet, the Weihenstephan strain generated a lower amount of respiratory deficient “petite” cells (not shown).

Lager yeast strains provide a clean taste to beers associated with rather low levels of aroma alcohols and esters compared to more fruity ale and wine yeasts. We used GC/FID to determine flavor differences in our group I and II lager yeast strains (Figure 2B and Table S2). *S. monacensis* produced only low amounts of flavors. Group I lager yeasts showed greater amounts of acetaldehyde (perceived as fruity at these concentrations) whereas the group II strain produced far more ethylacetate (pear drops flavor) and also more isoamyl alcohol/acetate (banana flavor).

### Sequencing of *S. carlsbergensis*

We sequenced the *S. carlsbergensis* genome using 454 GS FLX+ technology. More than 10^6^ reads were generated and assembled into a 19.5-Mb genome. We obtained >20× coverage with 680 bases average single-read length and an additional 8× coverage via an 8-kb paired end library with 325 bases average read length. A draft genome of the Weihenstephan has recently been generated (Nakao *et al.* 2009). Due to the large number of sequence contigs, we resequenced this strain using Illumina MiSeq v2 to obtain similar high level coverage and quality as for the *S. carlsbergensis* strain. To this end 10^7^ reads derived from 250-bp paired-end reads and an 8-kb mate-pair library were used and assembled in the 23 Mb genome resulting in a total coverage of 55x based on high quality reads (Table 1).

**Table 1:** Genome assembly data

	CBS1513	WS34/70
Number of contigs	386	1336
Number of scaffolds	78	985
N50 scaffold size	552,537	92,939
Largest scaffold size	1,262,187	1,252,108
Annotated bases in scaffolds	19,436,056	22,954,394

The Weihenstephan lager yeast contains essentially two complete parental genomes. As was shown previously, there is some loss of heterozygosity at chromosome ends, which in WS34/70 resulted in loss of ends of *S. eubayanus* chromosomes III, VII, XIII, and XVI (Nakao *et al.* 2009).

### Large-scale loss of *S. cerevisiae* parental DNA in *S. carlsbergensis*

**Figure 3:**
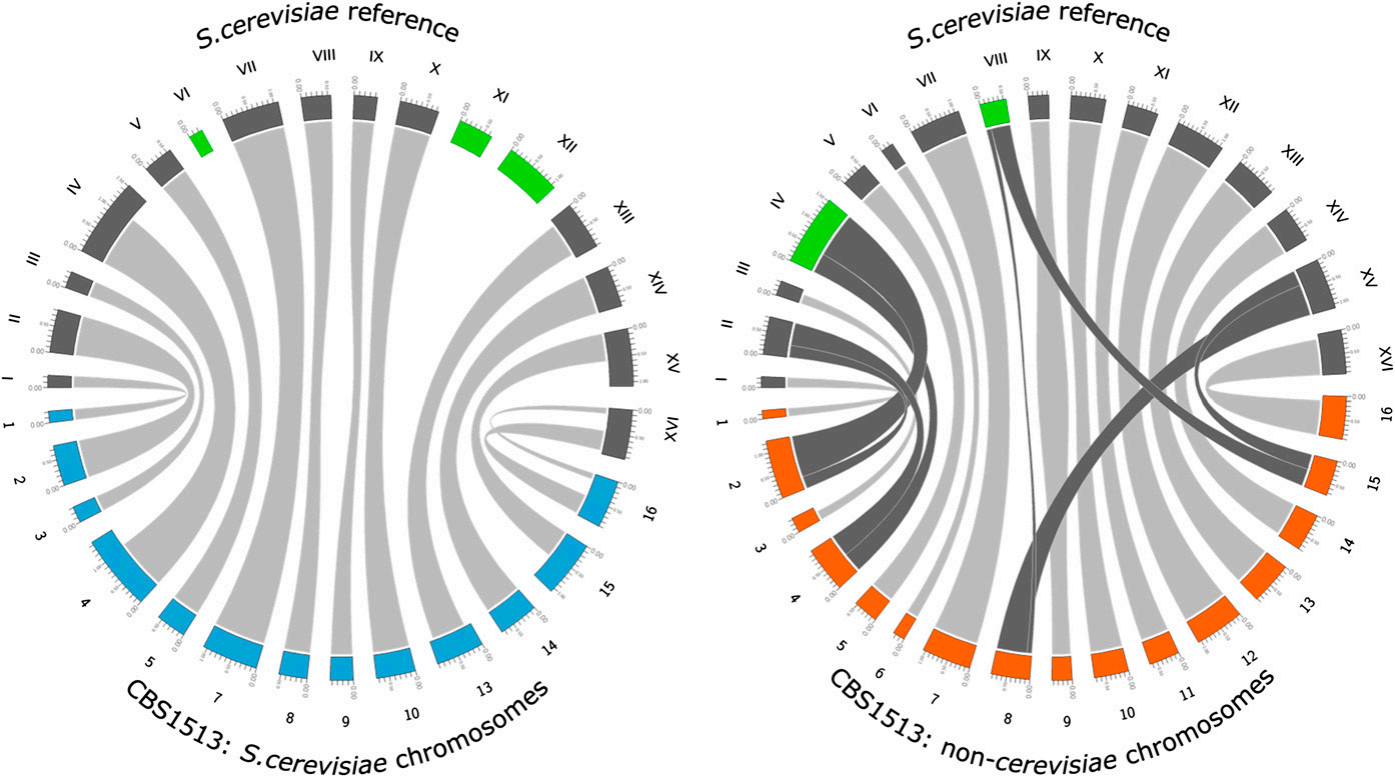
Overview of the *S. carlsbergensis* subgenomes. Genome sequencing data for *S. carlsbergensis* were assembled into the *S. cerevisiae* and *S. eubayanus* subgenomes and used in a pairwise comparison with the *S. cerevisiae* S288C chromosomes. The resulting information on all chromosome rearrangements is converted from tabular data into a circular plot by the Circos software package. Highlighted in green are the lack of chromosomes VI, XI, and XII in the *S. cerevisiae* sub-genome and the translocations between chromosomes II/IV and VIII/XV in the *S. eubayanus* sub-genome of *S. carlsbergensis* (see also Table 2).

We found a substantial size difference between WS34/70 and *S. carlsbergensis*, indicating a loss of app. 3.5 Mb from the group I lager yeast strain. To generate an overview of which parts of the parental genomes were lost, we partitioned the scaffolds into their *S. cerevisiae* and *S. eubayanus* origin based on sequence conservation. This is straightforward as scaffolds derived from the *S. cerevisiae* parent are >95% identical to the S288C genome sequence whereas scaffolds derived from the *S. eubayanus* parent are <95% identical to S288C. The two sets of scaffolds were then aligned to the S288C genome sequence. To generate a genome overview and visualize both parental genomes of *S. carlsbergensis* compared with the 16 *S. cerevisiae* chromosomes, we used CIRCOS (Figure 3; see the section *Materials and Methods* for details). It became apparent that *S. carlsbergensis* does not contain sequences from *S. cerevisiae* chromosomes VI, XI, and XII. Our results are consistent with previous data obtained by PCR-restriction fragment length polymorphism mapping or by array-CGH (Rainieri *et al.* 2006, Dunn and Sherlock 2008). Next to loss of complete *S. cerevisiae* chromosomes we identified several regions of loss of heterozygosity (LOH) in *S. cerevisiae* chromosomes IV, XIII, XV, and XVI (Figure 3 and Table 2). In contrast, there was only two position of LOH for the *S. eubayanus* part of chromosome III and XVI. In these cases sequences that were lost were replenished by orthologous regions from the other parental genome, which resulted in homozygous sequences derived from only one parental genome. In the *S. eubayanus* part two reciprocal translocations can be noted encompassing the chromosomes II and IV as well as VIII and XV (Figure 3). The total amount of DNA lost by chromosome loss and LOH is sufficient to explain the genome size difference between the group I and group II lager yeast strains. The Weihenstephan genome sequence initially was sized to 25 Mb. Our genome data comprises 23.6 Mb yet lacks telomeric regions due to redundancies. Thus, as indicated in Table 2, the difference between the *S. carlsbergensis* and Weihenstephan genomes can readily be explained by the loss of *S. cerevisiae* DNA observed in *S. carlsbergensis*.

**Table 2:** Genome reduction in *Saccharomyces carlsbergensis*

*S. cerevisiae* genome size, Mb	12.1
*S. bayanus* genome size, Mb	11.5
Hypothetical hybrid tetraploid genome, Mb	23.6
*Unterhefe* No. 1 genome, Mb	19.5
Loss of chromosomes	
CHR6	0.27
CHR11	0.67
CHR12	1.1
Loss of heterozygosity	
Chr3nonSc	0.11
CHR4sc	0.38
CHR13sc	0.12
CHR13sc	0.08
CHR15sc	0.48
CHR16nonSc	0.11
CHR16sc	0.36
CHR16sc	0.02
Total, Mb	23.2

### Generation of a chromosomal map of *S. carlsbergensis*

**Figure 4:**
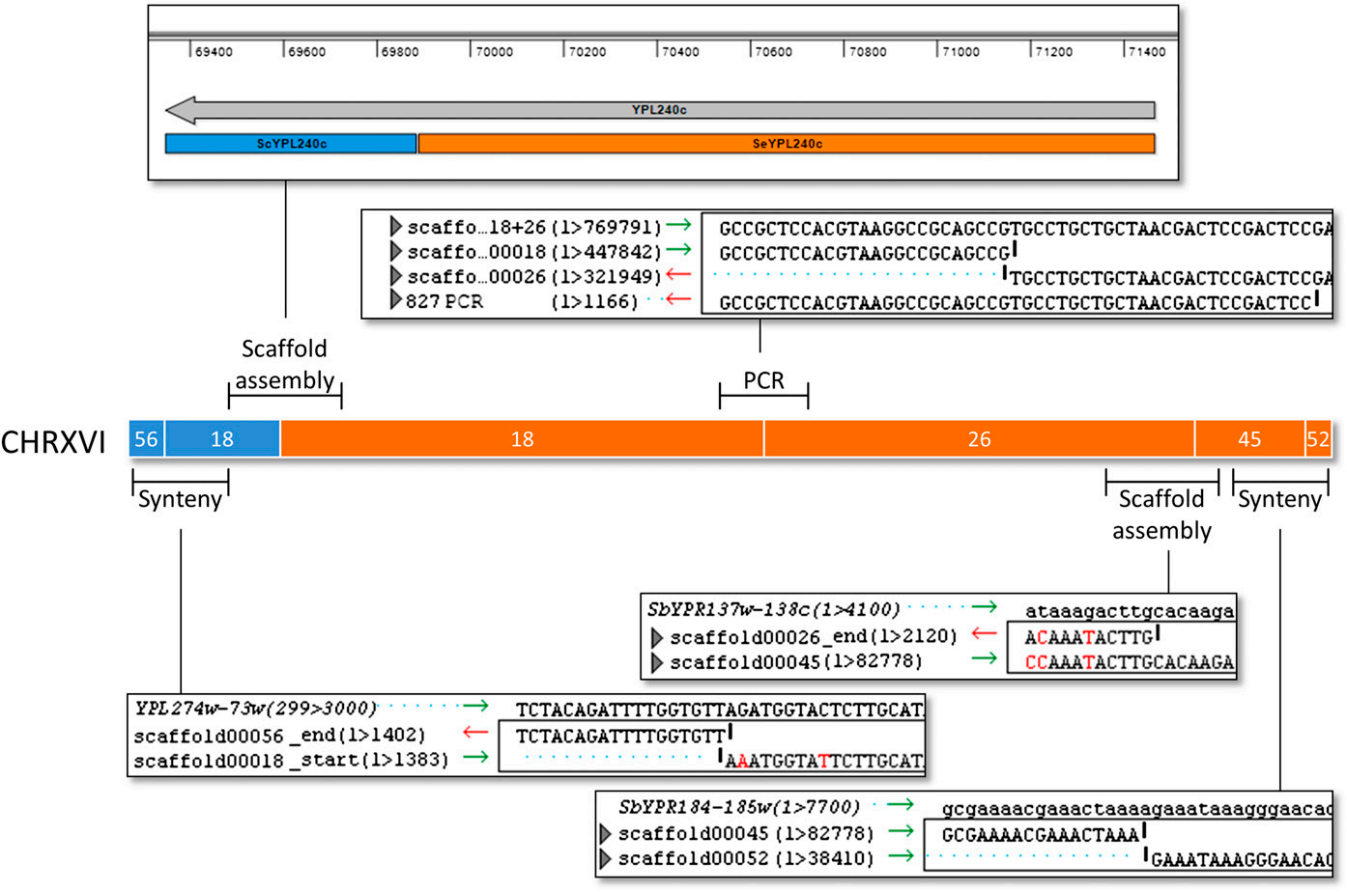
Strategy of chromosome assembly for the *S. carlsbergensis* genome. The scaffold composition of the chimeric chromosome XVI consisting of a telomeric left end derived from its *S. cerevisiae* parent and a major part of *S. eubayanus* is shown. Scaffolds were merged based on assembly of initial sequence reads (scaffold assembly) by directed polymerase chain reaction (PCR) using *S. eubayanus* specific primers to merge scaffolds 18 and 26 (PCR) or based on synteny to the parental genomes (synteny). Lasergene DNAstar software was used for sequence alignments, alignment snapshots of the scaffold ends and the linking PCR or template sequences are shown.

**Figure 5:**
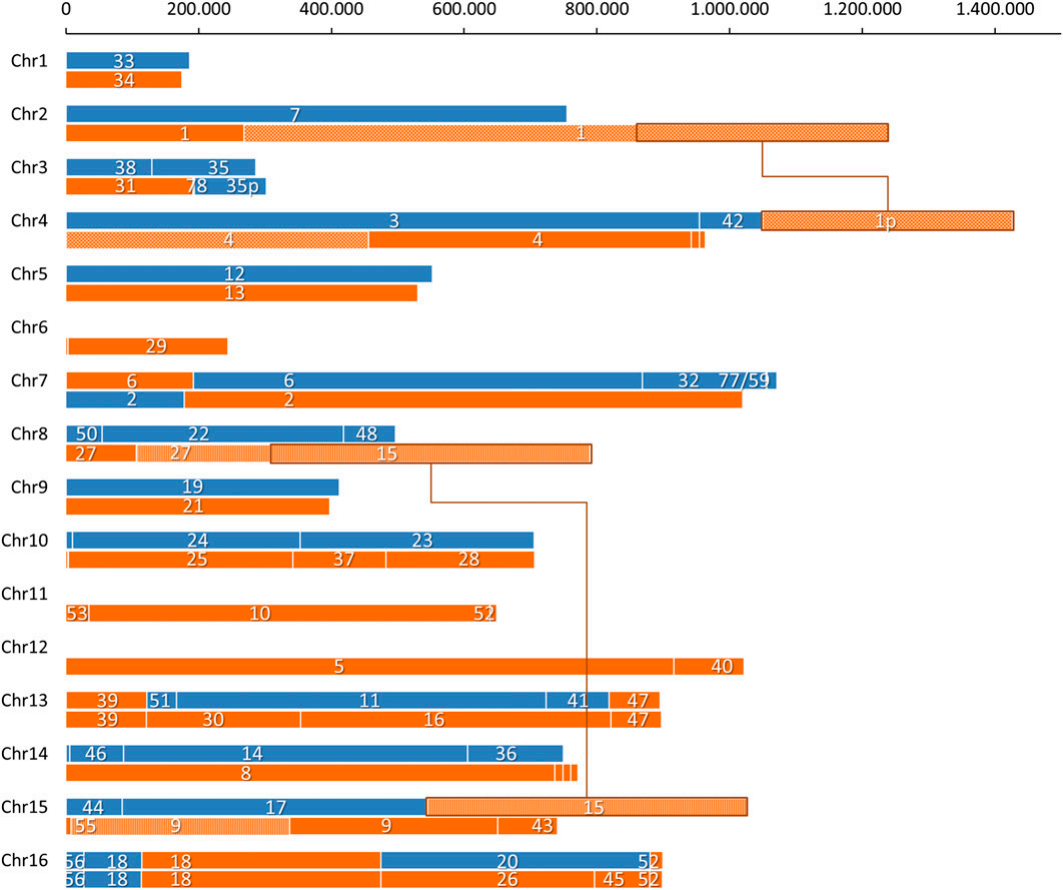
Chromosomal composition of the *S. carlsbergensis* genome. The chromosomal structure of the *S. carlsbergensis* genome is summarized and compared with the 16 chromosomes of the *S. cerevisiae* genome. The chromosome sizes are proportional to the size bar (in bp) on top of the chart. Chromosomal DNA derived from the *S. cerevisiae* subgenome is indicated in blue and that of the *S. eubayanus* subgenome in orange. Numbering corresponds to the scaffold numbers in the Genbank submission. Loss of heterozygosity, *e.g.*, on chromosomes III and XVI can be located, and reciprocal translocations in the *S. eubayanus* subgenome (II/IV and VIII/XV) are shown in checkers and stripes on the chromosomes, respectively. Additional loss of heterozygosity events in the *S. cerevisiae* subgenome on chromosomes IV and XV are marked with connectors encompassing the chromosomal segments involved.

Based on the high-quality sequencing we could assemble the *S. carlsbergensis* genome into just 78 scaffolds. Starting from these scaffolds we went on to merge the scaffolds into chromosome-size super scaffolds. There were basically two sets of scaffold breaks: either within parental scaffolds (Sc/Sc or Se/Se) or in case of LOH and lack of contiguous sequences between Sc/Se scaffolds (Table 3). We used directed PCR sequencing to obtain evidence of scaffold linkages. As an example the hybrid chromosome XVI is shown (Figure 4). Preliminary assembly of this chromosome containing five scaffolds was done based on synteny to *S. cerevisiae*. One scaffold, scaffold 18, covered the position of a reciprocal translocation within YPL240C of the *S. cerevisiae* and *S. eubayanus* parental genomes. Two scaffolds, 26 and 45, could be manually assembled and were initially separated due to their short overlapping regions. Scaffolds 18 and 26 were joined by directed PCR using primers specific for the *S. eubayanus* sequence. The remaining two gaps were located between Sc/Sc and Se/Se scaffolds and were joined based on synteny (Figure 4). We analyzed all scaffolds in this way. Some scaffolds were merged based on synteny with their gaps presumably marking positions of transposable elements (Table S3). Linkage mapping of all scaffolds resulted in a total of 29 different chromosomes for *S. carlsbergensis* (Figure 5). In contrast, the Weihenstephan lager yeast was shown to harbor 36 different chromosomes, which is confirmed by our analysis (Nakao *et al.* 2009). The complete list of *S. carlsbergensis* chromosomes also enables an overview of the number and position of translocations in this genome (Table 4). In the *S. carlsbergensis* genome we find conserved reciprocal translocations between the *S. eubayanus*-derived chromosomes II/IV and VIII/XV that are also present in the WS34/70 strain. Apparently, these translocations are ancestral as they also occur in *S. bayanus*. Interestingly, the *S. carlsbergensis* and Weihenstephan lager yeast strains share three translocations: One on chromosome XVI shown in Figure 4 within YPL240C, another one at the *MAT*-locus, and the last one within YGL173C. In addition *S. carlsbergensis* harbors seven unique translocations between chromosomes of both parental genomes. The translocations generated several chimeric chromosomes that are a distinguishing feature of this yeast. WS34/70, on the other hand, carries eight translocations that are specific for this strain.

**Table 3:** Chromosomal make-up of *Saccharomyces carlsbergensis*

Sc Chr	SC Copies	Chimeric	Se Chr	Se Copies	Unique Chr
I	1	0	I	1	2
II	1	0	II-IV	2	2
III	2	1	III	0	2
IV	0	1	IV-II	2	2
V	1	0	V	2	2
VI	0	0	X-VI	3	1
VII	0	3*^a^*	VII	0	2
VIII	1	0	VIII-XV	2	2
IX	1	0	IX	2	2
X	1	0	VI-X	2	2
XI	0	0	XI	3	1
XII	0	0	XII	3	1
XIII	0	1	XIII	2	2
XIV	1	0	XIV	2	2
XV	0	1	XV-XIII	2	2
XVI	0	3*^a^*	XVI	0	2
Sum	9	10		28	29
Total	47				

Se, *S. eubayanus*; Chr, chromosome.

a For Chr7 and 16 are 2 different chimera present.

**Table 4:** List of translocations in the *Saccharomyces carlsbergensis* genome

Type	Translocation	Gene: Systematic Name	Gene: Standard Name
S.eub-S.eub	II-IV	**YBR030w-YDR012w***^a^*	RKM3-RPL4B
	IV-II	**YDR011w-YBR031w***^a^*	SNQ2-RPL4A
	VIII-XV	**YHR014w-YOR019w***^a^*	SPO13-n/a
	XV-VIII	**YOR018w-YHR015w***^a^*	ROD1-MIP6
S.eub-S.cer	III-III	**YCR038c-YCR039c***^a^*	BUD5-MATALPHA2
	VII-VII	**YGL173c-YGL173c***^a^*	XRN1
	XIII-XIII	YML074c-YML073c	FPR3-RPL6A
	XVI-XVI	YPL036c-YPL036c	PMA2
S.cer-S.eub	IV-IV	YDR324c-YDR324c	UTP4
	VII-VII	YGL173c-YGL173c	KEM1
	XIII-XIII	YMR287c-YMR287c	MSU1
	XV-XV	YOR133w-YOR134w	EFT1-BAG7
	XVI-XVI	**YPL240c-YPL240c***^a^*	HSP82
	XVI-XVI	YPR184w-YPR185w	GDB1-ATG13

a Indentical translocations to WS34/70.

### Ploidy assessment for *S. carlsbergensis*

**Figure 6:**
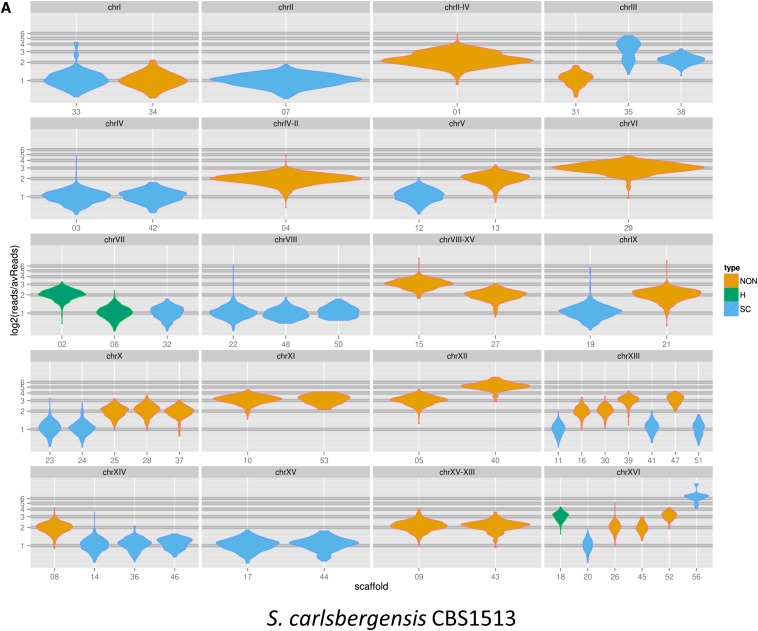

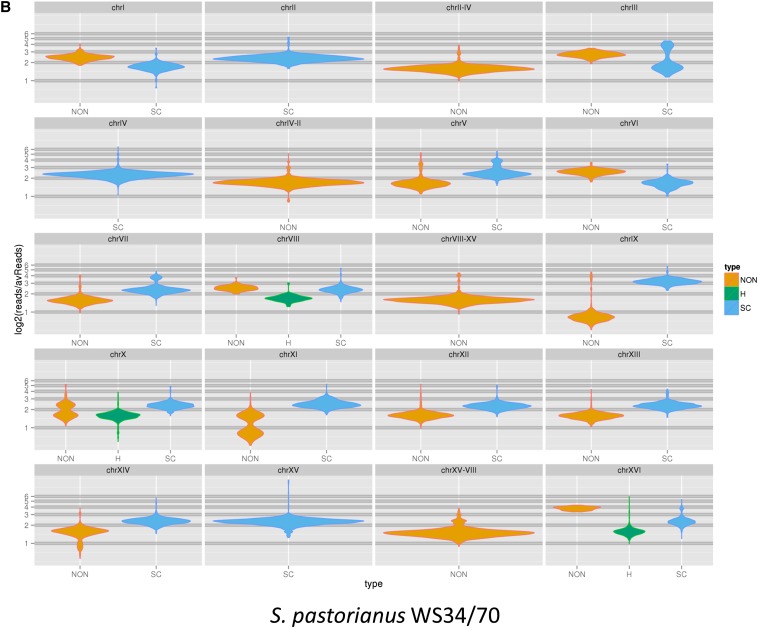

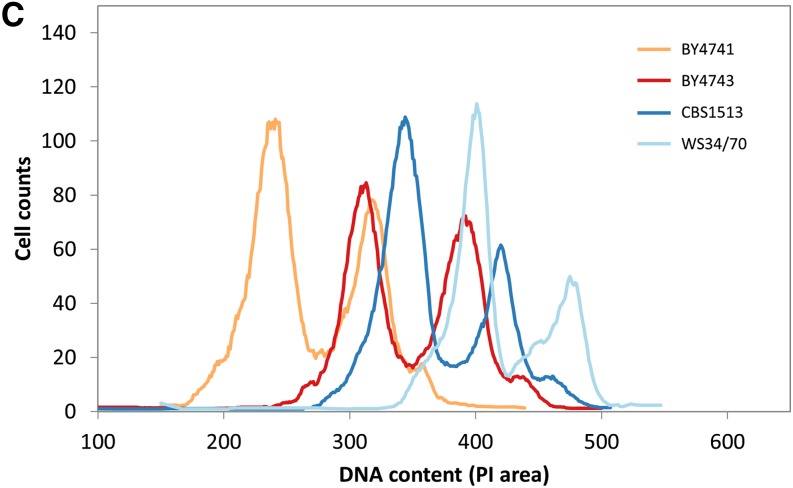
Ploidy analysis for the *S. carlsbergensis* genome. A violin plot generated by ggplot2 shows the ploidy calculated based on the read depth for *S. carlsbergensis* CBS1513 (A) and *S. pastorianus* WS34/70 (B). The individual reads were aligned to the respective scaffolds using LASTZ, the alignment output was parsed with SAMtools in order to index and extract the number of aligned reads at each locus. A running average of mapped reads per 1500 bp window was calculated. The violin plot shows the distribution of the log2 ratios of copy number variation across each chromosome. Blue represents the *S. cerevisiae* sub-genome, orange the *S. eubayanus* sub-genome and green indicates hybrid scaffolds/chromosomes. (C) Flow cytometry analysis of the DNA content of the *S. carlsbergensis* and Weihenstephan strains compared to 1n and 2n laboratory strains. DNA content is plotted *vs.* cell counts.

**Figure 7:**
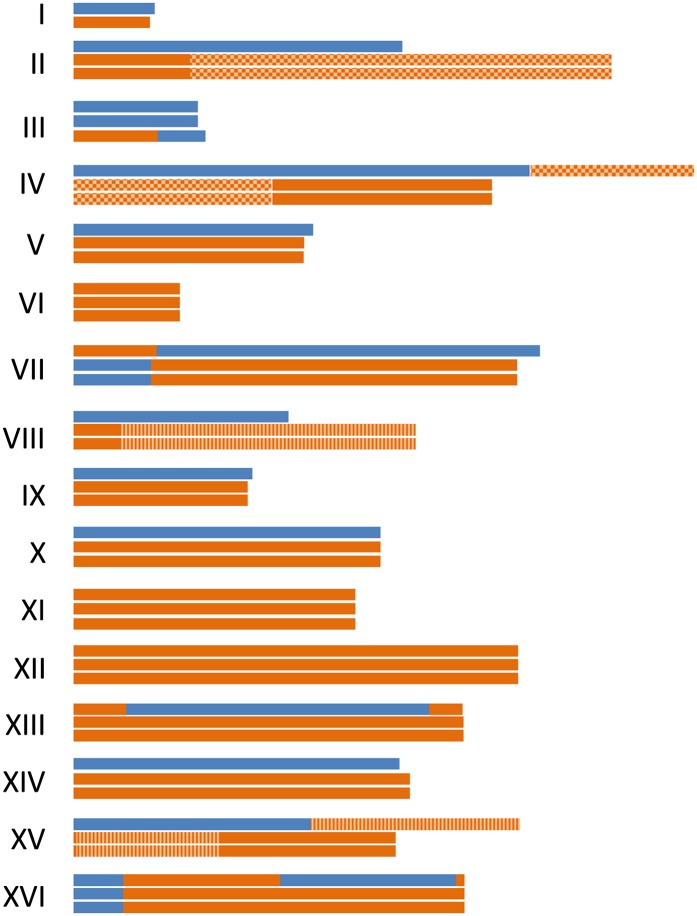
Chromosomal structure and chromosome copy number of *S. carlsbergensis*. The 16 *Saccharomyces sensus stricto* chromosomes were used to align the chromosomes of *S. carlsbergensis*. Blue parts of chromosomes represent the *S. cerevisiae* sub-genome, orange the *S. eubayanus* sub-genome. Ploidy was taken into account to represent the parental contribution.

Previous reports suggested that based on array hybridization signal intensities group I lager yeasts have a DNA content resembling that of 2n *S. cerevisiae*. Thus it was hypothesized that group I lager yeasts originated from a hybridization of two haploid *S. cerevisiae* and *S. eubayanus* cells (Dunn and Sherlock 2008). Based on the high coverage sequencing, we could use the amount of sequence reads per scaffold unit size as a measure of abundance of the corresponding chromosomes. To this end we used LASTZ to map all *S. carlsbergensis* reads to the scaffolds and SAMtools to get sorted alignments of the reads to the scaffolds. This was used to calculate the read depth per 1500 bp window. The data were visualized using ggplot2 in R (Figure 6A). The data were consistent with our mapping of individual scaffolds into chromosomes and the additional translocation events observed, *e.g.*, based on our chromosomal map the *S. eubayanus*-derived scaffold 15 is present in three copies. Based on these data, we were able to generate an overview on the ploidy of each chromosome in *S. carlsbergensis* (Figure 7 and Table 3). Surprisingly, these results indicate that *S. carlsbergensis* basically is a triploid strain harboring less than one haploid *S. cerevisiae* and more than a diploid *S. eubayanus* genome. Two chromosomes are distinct: chromosome I is present only in one *S. cerevisiae* and one *S. eubayanus* copy each, whereas chromosome III is present in two *S. cerevisiae* copies and only one *S. eubayanus* copy. Based on our data, we can infer that *S. carlsbergensis* harbors 29 different chromosomes and a total of 47 chromosomes, *i.e.*, 3n-1 (Table 3). Of these 47 chromosomes, 10 are chimeric. The ploidy analysis also revealed that the ratio for *S. eubayanus vs. S. cerevisiae* derived DNA is 2:1 in *S. carlsbergensis* (Figure 6A). A similar analysis for WS34/70 using our sequence data indicates that this group II lager yeast strain is tetraploid composed of basically two diploid *S. cerevisiae* and *S. eubayanus* genomes (Figure 6B). We also performed FACS analyses with both lager yeast strains to obtain independent evidence on their ploidies compared with 1n and 2n laboratory strains (Figure 6C). These data are consistent with the ploidy data generated from NGS data.

## Discussion

The tradition of beer making is ancient and goes back thousands of years. In central Europe, it was associated with monks, and one of the most famous places was Munich in Bavaria (which actually literally means “the monks’ place”). Originally dark beers were produced that became replaced at the end of the 19th century with pilsner-type beers. This movement may have originated in Munich, as Gabriel Sedlmayer started pale ale production in the Spaten Brewery of Munich (Hornsey 2003; Kodama *et al.* 2005). From there, lager beer brewing spread to Pilsen, Czech Republic, and to Copenhagen, Denmark. This geographic associations and affiliations with specific breweries is reflected in today’s grouping of lager yeasts as group I (Czech and Carlsberg) or group II (Weihenstephan and Heineken) (Dunn and Sherlock 2008).

Lager beer production was revolutionized by the use of pure culture lager yeast strains introduced by Emil Chr. Hansen at Carlsberg (Hansen 1883). Up to this time point, new brews were initiated by repitching yeasts from a previous brew. This tradition has been kept; however, from then on care was taken that the production strains were kept isolated from other yeast strains.

Here we have determined the genome sequence of *Saccharomyces carlsbergensis*, the Carlsberg brewery production strain since 1883. Our data fit well with previous reports either covering the hybrid nature of lager yeasts based on the study of single genes or using more global analyses like array-CGH (Rainieri *et al.* 2006, Dunn and Sherlock 2008).

A clear distinction between group I and group II lager yeast strains is the selective loss of parts of the *S. cerevisiae* parental genome in group I lager yeasts. For *S. carlsbergensis* this resulted in the complete loss of three chromosomes (VI, XI, and XII) and in loss of heterozygosity at four chromosomes (IV, XIII, XV, and XVI) amounting to a total of >3.5 Mb of *S. cerevisiae* DNA. The driving force behind this evolution is yet unclear. Loss of chromosome 12, for example, encompasses elimination of the *S. cerevisiae* ribosomal (r)DNA cluster in *S. carlsbergensis*. In contrast in the Weihenstephan strain, a massive loss of the *S. eubayanus* rDNA cluster was observed (Nakao *et al.* 2009). The loss of other genome parts may be attributed to the cold fermentation conditions applied during lager beer fermentation in the 19th century. At Carlsberg, fermentation temperatures were as low as 5°. We found, correspondingly, that *S. carlsbergensis* was better adapted to cold temperature growth conditions than group II lager yeasts, including the WS34/70 strain. The *S. eubayanus* ancestor is also a psychrophilic strain; thus, maintenance of the *S. eubayanus* genome part may have been selected for under these fermentation conditions. Current lager beer fermentations are carried out at considerably greater temperatures and *e.g.*, at 14° the group II lager yeasts are slightly faster than group I strains (see Figure 2; Saerens *et al.* 2008).

In *S. carlsbergensis* we noted loss of the left arm of *S. eubayanus* chromosome XVI (~100 kb) and loss of the right arm of chromosome III starting at the *MAT*-locus (~100 kb). Both positions involved translocations between the respective *S. cerevisiae* and *S. eubayanus* chromosomes and, interestingly, are conserved between group I and group II lager yeasts. Genes involved in these translocations are the *MAT*-locus and the *HSP90* homolog *HSP82*. These rearrangements may have played key roles in lager yeast evolution. Alterations at the *MAT*-locus may have been instrumental to, *e.g.*, avoid sporulation under adverse conditions such as at the end of fermentation. The translocation at *HSP82* occurred within the gene and thus has generated a chimeric gene. *HSP82*, encodes a *HSP90* chaperone required, *e.g.*, for refolding of denatured proteins (Burnie *et al.* 2006; Pursell *et al.* 2012). *HSP90* has been shown to act as a capacitor for morphological evolution (Rutherford and Lindquist 1998; Taipale *et al.* 2010). Thus, a translocation at YPL240C may have played a substantial role in lager yeast evolution that is currently under further investigation.

Genome sequencing in lager yeasts is only at its early beginnings and the *S. carlsbergensis* genome presented here is the first done using next-generation sequencing technologies, which we also used to update the genome sequence of the WS34/70 strain. Several hypotheses have been developed on the evolution of lager yeast and the origin of the parental yeast strains. Currently, the non-*cerevisiae* parent is viewed to be a close relative of *S. eubayanus*, and the *S. cerevisiae* parent may have been a strain already used for beer brewing, *e.g.*, an ale yeast (Dunn and Sherlock 2008; Bond 2009; Libkind *et al.* 2011; Nguyen *et al.* 2011). Our work adds to this as we can promote two hypotheses. First, based on our ploidy analyses, *S. carlsbergensis* is functionally an allotriploid strain, whereas group II lager yeasts are allotetraploid. This could argue in favor of at least two independent hybridization events in that group I lager yeasts were generated by a fusion of 1n *S. cerevisiae* with 2n *S. eubayanus* and group II lager yeasts by fusion of two 2n yeasts. However, comparison of translocations in *S. carlsbergensis* with those in the Weihenstephan strain identified three conserved events and seven to eight strain specific events. Based on these conserved events, however, and based on the history of lager beer production originating in Munich, we favor the notion that both strains share a joint history and a common ancestor. Previously, a close relationship of CBS1513 with WS34/70 was also proposed based on the analysis of single genes (Nguyen *et al.* 2011). A joint ancestry, on the other hand, suggests that *S. carlsbergensis* evolved by massively reducing its *S. cerevisiae* genome content. Most of this evolution was apparently due to whole chromosome loss but could also have come about by meiotic reduction and remating. Whole-genome duplication in the shared ancestor between *S. cerevisiae* and, *e.g.*, *S. castellii*, was also followed by massive gene losses. In *S. cerevisiae* this did not lead to wholesale chromosome loss, whereas *S. castellii* has reduced the number of chromosomes from 16 to 9 (Cliften *et al.* 2006; Scannell *et al.* 2007). We are currently investigating this in more detail by, *e.g.*, by comparing the relationship between *S. carlsbergensis* and *S. monacensis*, another strain isolated by Hansen in the 19th century, which apparently has lost additional parts of the *S. cerevisiae* parental genome (our unpublished data).

The use and conservation of pure culture yeast strains in lager beer production has had a profound impact on the quality and reproducibility in beer production and promoted large scale productions. Yet, at the same time evolution of lager yeast strains was impaired under these conditions. Batches of beer that were inferior compared with the standard or became contaminated were readily discarded as the production strain could be propagated from a pure culture. This generated consistent production results and on the other hand diminished the chance of yeast strains to evolve further. To estimate the level of diversity, we obtained several historic bottles from the Carlsberg Museum bottle collection filled with original beer from the late 19th century. In the slurry present in these bottles yeast cells were found that could be stained with the cell wall dye calcofluor white. Using PCR we could detect *S. carlsbergensis*−specific DNA fragments. Attempts to isolate living material from these bottles generated two isolates, one determined as *Sporobolomyces roseus* (a beer spoilage yeast not present in our lab) and the other as *S. carlsbergensis* based on rDNA sequencing. Sequencing of this presumptive bottle isolate revealed it to be identical to the CBS1513 genome sequence, which has been kept in the CBS strain collection since 1947 with minimal propagation cycles. This suggests very limited evolution of pure cultured yeast strains under industrial fermentation conditions. Future work on lager yeast evolution will not only cover the historic spectrum of lager yeast strains but move on to demonstrate the evolutionary potential of lager yeast hybrids to adapt to altered fermentation conditions and to study the dynamics within lager yeast populations.

## 

## Supplementary Material

Supporting Information
