# Supplementary material for: Genome Sequence of Saccharomyces carlsbergensis, the World’s First Pure Culture Lager Yeast
Source: G3 (Bethesda). 2014 Feb 27;4(5):783–93. doi: 10.1534/g3.113.010090 (PMC4025477; doi:10.1534/g3.113.010090)
Supplement: Supporting Information [file supp_4_5_783__index.html]

Genome Sequence of Saccharomyces carlsbergensis, the World’s First Pure Culture Lager Yeast — Supporting Information 

# Genome Sequence of *Saccharomyces carlsbergensis*, the World’s First Pure Culture Lager Yeast

## Supporting Information for Walther, Hesselbart, and Wendland, 2014

**Files in this Data Supplement:**

- Supporting Information - Tables S1-S3 (PDF, 163 KB)
- Table S1 - Primers used in this study to bridge gaps between scaffolds. (PDF, 81 KB)
- Table S2 - Volatile compound analysis (PDF, 80 KB)
- Table S3 - Scaffold assembly for *S. carlsbergensis* (PDF, 146 KB)
