# Supplementary material for: Genome Sequence of Saccharomyces carlsbergensis, the World’s First Pure Culture Lager Yeast
Source: G3 (Bethesda). 2014 Feb 27;4(5):783–93. doi: 10.1534/g3.113.010090 (PMC4025477; doi:10.1534/g3.113.010090)
Supplement: Supporting Information [file supp_g3.113.010090_010090SI.pdf]

G3 · Genes | Genomes | Genetics

Genome sequence of *Saccharomyces carlsbergensis,* the world's first pure culture lager yeast

Andrea Walther, Ana Hesselbart and Jürgen Wendland*

Carlsberg Laboratory, Yeast Biology, Gamle Carlsberg Vej 10, Valby, Denmark

(*) Corresponding author: Jürgen Wendland, Carlsberg Laboratory, Yeast Biology, Gamle Carlsberg Vej 10, DK-1799 Copenhagen V, Denmark

Email: juergen.wendland@carlsberglab.dk
Phone: +45/3327 5230;
Fax: +45/3327 4708

DOI: 10.1534/g3.113.010090

Table S1 Primers used in this study to bridge gaps between scaffolds

Chr	Primer 1	sequence	Primer 2	sequence 2	scaffold
2/4	6479	GGCGACAGCACGACCAGTACCCC	6485	GGAAGCAGGAGTGACTGAGGC	1
4	6591	GTTGCGTGTGCACAGTAGAGGG	6611	cagtgtactccggaggagcattcac	3+42
4	6964	GTGTAATAGACGACCCATG	6965	GAAGGCTTATGCTGGAGC	42+1p
4/2	6480	GGTATGTAAGTAAAACCAG	6486	CATAAGGAAAAGTAAGCAATG	4
7	6482	CTCTTAATAAAGAAAGGTGCAAAAGAA	6488	TAGTCCACCTGTACTTGGGCCG	6
7	6593	CTGATTCATAGCCTCGTAtAG	6613	GCTCAGAGTTTACCAAGAGAG	6+32
7	6481	CTTAATAAAGAAAGATGTAATAAGT	6487	GTTGTTCCAGGCATACTCGCA	2
7	6594	CCAGTAGGTCCTCTGTAAGAC	6614	CCAATGTCGACCCAGACGAG	32+77
7	6595	CAACCACCAGATTTGATGTTC	6615	GAGATTCGCCAAGCAGACGTC	77+59
8	6596	CATAAATTTGATCTTAGCATC	6616	GGACAACGTGGATAGCAAGGAC	50+22
8/15	6520	GTTATTTCGTTCGGTTCTCAAGG	6528	CTGTAAGGATTATTTGGTCAAACTC	27+15
8/15	6551	CTGTGATCCAAAGGCTGACTTG	6552	GTATACTACCTTATTATATGTATTAC	27+15
10	6598	GGAGAATATTGACAGTTACAC	6618	GTGAAAGTTCTTTGCAAGATG	24+23
10	6599	GGATTGCAGACCATTTCATC	6619	GAATTTCGTTATTCCTACCTTC	25+37
10	6600	CAGATTCTCCAAATTACTGG	6620	GAAGAAGCTGCCATTTCCG	37+28
11	6601	CTCGAAGACAGTTCATCGTATG	6621	CTGTGTGTTCCGTAGTTCACC	53+10
12	6602	GATGACCGTAAATGCTGCTTG	6622	CGAACGACTTGTCAGACGC	5+40
13	6517	GGTGATACGGATGGTAATGGGCATCG	6526	CATTGCTACCTCTACTAAGGTT	39+51
13	6604	CTGGTGGGGCTGTGTGGATGC	6624	CTACCCGTAATCAAGTTGCC	11+41
13	6515	GCAAGTAATTCCAATATTAATGG	6523	CTATTGGGTGTACCGAGTATCCTG	41+47
13	6517	GGTGATACGGATGGTAATGGGCATCG	6525	CATTGCCACCTCCACCAACGTC	39+30
13	6548	CCGTTCTACCCTGAACTACGAG	6549	TCACGACTTATAGCTGCAATTAATCAC	39+30
13	6605	CCATTGTTGACCTGATTCTG	6625	GATGAAGGCCTTCGACCGTC	30+16
13	6515	GCAAGTAATTCCAATATTAATGG	6524	CTTTATACGTCGCTCCCTCAG	16+47
14	6606	CAGCGAAGTGGAAGATGACC	6626	GAACATCAAGGCTTTGGACC	46+14
14	6607	GACATTGTCTTGTGGAGCC	6627	GCAATGATAAGGTGTTCTTC	14+36
15	6608	GACGAAGACAGTTAGCTTTAC	6628	GAGCGGACCATTGCAAGTGG	44+17
15	6519	gtcgctgtcgaagtcaagaacgc	6527	GTTGGGGACGAGTCGCCTTGAG	17+15
15	6550	CAGAATCCAAGGTCCAAACTACG	6528	CTGTAAGGATTATTTGGTCAAACTC	17+15
15/8	6609	GCCATCTCATTTAGCACCAGC	6629	GTCACCATCTCTCATTTCCAAG	9+43
16	6521	GCACCAGCAGAAGAGTTAGTC	6529	TGAGTCAGAATCATCAGCGGCAGAAG	18+20
16	6521	GCACCAGCAGAAGAGTTAGTC	6530	GGAGTCGGAGTCGTTAGCAGCAGGCA	18+26
16	6522	GAAAGTGTGTGGGCAGGATTG	6531	CTCTTTCTCGTTATTCAGATTG	20+52

Table S2 Volatile compound analysis

compound		WS34/70			CBS1513			CBS1503		
Ethanol	% v/v	5,86	±	0,01	6,04	±	0,01	5,13	±	0,00
acetaldehyde	ppm	3,5	±	0,7	5,2	±	0,2	4,5	±	0,2
ethylacetate	mg/l	35,1	±	1,3	18,1	±	1,3	17,5	±	4,4
isobutanol	mg/l	116,8	±	11,0	194,9	±	1,4	76,6	±	27,2
isobutylacetate	mg/l	0,3	±	0,1	0,2	±	0,1	0,1	±	0,1
propanol	mg/l	31,0	±	1,1	25,4	±	0,3	24,8	±	1,2
isoamyl alcohol	mg/l	244,1	±	17,2	224,3	±	1,2	174,0	±	32,1
isoamylacetate	mg/l	7,5	±	0,8	4,9	±	0,4	2,5	±	1,1
2-phenylethanol	mg/l	57,8	±	6,4	76,9	±	7,2	60,1	±	17,9
2-phenylethyl acetate	mg/l	1,3	±	0,1	1,3	±	0,1	0,7	±	0,3
ethyl hexanoate	mg/l	0,2	±	0,1	0,2	±	0,0	0,2	±	0,1
ethyl octanoate	mg/l	0,2	±	0,0	0,2	±	0,0	0,1	±	0,0
hexanoic acid	mg/l	0,8	±	0,0	0,7	±	0,1	0,7	±	0,2
octanoic acid	mg/l	5,4	±	0,2	6,5	±	0,3	5,6	±	1,6
vinyl gaïacol	mg/l	0,7	±	0,1	0,7	±	0,1	0,8	±	0,0
Decanoic acid	mg/l	0,2	±	0,0	1,3	±	0,1	1,3	±	0,3
Total acids	mg/l	6,4	±	0,2	8,5	±	0,3	7,6	±	1,6
Sub-total alcohols	mg/l	205,6	±	17,8	297,3	±	8,8	161,4	±	44,7
Total esters	mg/l	43,3	±	2,2	23,7	±	1,8	20,3	±	5,5

Table S3 Scaffold assembly for *S. carlsbergensis*

Saccharomyces carlsbergensis			
Sc Chr	Scaffolds	Se Chr	Scaffolds
I	33	I	34
II	7	II-IV	1
III	38 (TY) 35	III	31+78+35part
IV	3+42+1part	IV-II	4
V	12	V	13
VI	-	VI	29
VII	6+32+77+59	VII	2
VIII	50+22**48	VIII-XV	27+15
IX	19	IX	21
X	24 (TY) 23	X	25*37+28
XI	-	XI	53*10, 52
XII	-	XII	5+40
XIII	39+51 (TY) 11+41+47	XIII	39+30 (TY) 16+47
XIV	46 (TY) 14 (TY) 36	XIV	8
XV	44+17+15	XV-XIII	9+43
XVI	56+18*20+52	XVI	56+18+26+45+52

A “+” indicates that scaffolds were combined by PCR and sequencing. Grey boxes mark scaffolds that represent either chromosomes consisting of only one scaffold or chromosomes which were generated by merging scaffolds. Red boxes mark scaffolds with gaps containing TY-elements. A “*” indicates a gap < 0.5 kb verified by PCR and “**” indicates the region between YHR165C and YHR174W that is apparently missing in *S. carlsbergensis*.
