# Supplementary material for: Genome Sequence of Saccharomyces carlsbergensis, the World’s First Pure Culture Lager Yeast
Source: G3 (Bethesda). 2014 Feb 27;4(5):783–93. doi: 10.1534/g3.113.010090 (PMC4025477; doi:10.1534/g3.113.010090)
Supplement: Supporting Information [file supp_g3.113.010090_TableS1.pdf]

**Table S1 Primers used in this study to bridge gaps between scaffolds**

| Chr | Primer 1 | sequence | Primer 2 | sequence 2 | scaffold |
|---|---|---|---|---|---|
| 2/4 | 6479 | GGCGACAGCACGACCAGTACCCC | 6485 | GGAAGCAGGAGTGACTGAGGC | 1 |
| 4 | 6591 | GTTGCGTGTGCACAGTAGAGGG | 6611 | cagtgtactccggaggagcattcac | 3+42 |
| 4 | 6964 | GTGTAATAGACGACCCATG | 6965 | GAAGGCTTATGCTGGAGC | 42+1p |
| 4/2 | 6480 | GGTATGTAAGTAAAACCAG | 6486 | CATAAGGAAAAGTAAGCAATG | 4 |
| 7 | 6482 | CTCTTAATAAAGAAAGGTGCAAAAGAA | 6488 | TAGTCCACCTGTACTTGGGCCG | 6 |
| 7 | 6593 | CTGATTCATAGCCTCGTAtAG | 6613 | GCTCAGAGTTTACCAAGAGAG | 6+32 |
| 7 | 6481 | CTTAATAAAGAAAGATGTAATAAGT | 6487 | GTTGTTCCAGGCATACTCGCA | 2 |
| 7 | 6594 | CCAGTAGGTCCTCTGTAAGAC | 6614 | CCAATGTCGACCCAGACGAG | 32+77 |
| 7 | 6595 | CAACCACCAGATTTGATGTTC | 6615 | GAGATTCGCCAAGCAGACGTC | 77+59 |
| 8 | 6596 | CATAAATTTGATCTTAGCATC | 6616 | GGACAACGTGGATAGCAAGGAC | 50+22 |
| 8/15 | 6520 | GTTATTTCGTTCGGTTCTCAAGG | 6528 | CTGTAAGGATTATTTGGTCAAACTC | 27+15 |
| 8/15 | 6551 | CTGTGATCCAAAGGCTGACTTG | 6552 | GTATACTACCTTATTATATGTATTAC | 27+15 |
| 10 | 6598 | GGAGAATATTGACAGTTACAC | 6618 | GTGAAAGTTCTTTGCAAGATG | 24+23 |
| 10 | 6599 | GGATTGCAGACCATTTCATC | 6619 | GAATTTCGTTATTCCTACCTTC | 25+37 |
| 10 | 6600 | CAGATTCTCCAAATTACTGG | 6620 | GAAGAAGCTGCCATTTCCG | 37+28 |
| 11 | 6601 | CTCGAAGACAGTTCATCGTATG | 6621 | CTGTGTGTTCCGTAGTTCACC | 53+10 |
| 12 | 6602 | GATGACCGTAAATGCTGCTTG | 6622 | CGAACGACTTGTCAGACGC | 5+40 |
| 13 | 6517 | GGTGATACGGATGGTAATGGGCATCG | 6526 | CATTGCTACCTCTACTAAGGTT | 39+51 |
| 13 | 6604 | CTGGTGGGGCTGTGTGGATGC | 6624 | CTACCCGTAATCAAGTTGCC | 11+41 |
| 13 | 6515 | GCAAGTAATTCCAATATTAATGG | 6523 | CTATTGGGTGTACCGAGTATCCTG | 41+47 |
| 13 | 6517 | GGTGATACGGATGGTAATGGGCATCG | 6525 | CATTGCCACCTCCACCAACGTC | 39+30 |
| 13 | 6548 | CCGTTCTACCCTGAACTACGAG | 6549 | TCACGACTTATAGCTGCAATTAATCAC | 39+30 |
| 13 | 6605 | CCATTGTTGACCTGATTCTG | 6625 | GATGAAGGCCTTCGACCGTC | 30+16 |
| 13 | 6515 | GCAAGTAATTCCAATATTAATGG | 6524 | CTTTATACGTCGCTCCCTCAG | 16+47 |
| 14 | 6606 | CAGCGAAGTGGAAGATGACC | 6626 | GAACATCAAGGCTTTGGACC | 46+14 |
| 14 | 6607 | GACATTGTCTTGTGGAGCC | 6627 | GCAATGATAAGGTGTTCTTC | 14+36 |
| 15 | 6608 | GACGAAGACAGTTAGCTTTAC | 6628 | GAGCGGACCATTGCAAGTGG | 44+17 |
| 15 | 6519 | gtcgctgtcgaagtcaagaacgc | 6527 | GTTGGGGACGAGTCGCCTTGAG | 17+15 |
| 15 | 6550 | CAGAATCCAAGGTCCAAACTACG | 6528 | CTGTAAGGATTATTTGGTCAAACTC | 17+15 |
| 15/8 | 6609 | GCCATCTCATTTAGCACCAGC | 6629 | GTCACCATCTCTCATTTCCAAG | 9+43 |
| 16 | 6521 | GCACCAGCAGAAGAGTTAGTC | 6529 | TGAGTCAGAATCATCAGCGGCAGAAG | 18+20 |
| 16 | 6521 | GCACCAGCAGAAGAGTTAGTC | 6530 | GGAGTCGGAGTCGTTAGCAGCAGGCA | 18+26 |
| 16 | 6522 | GAAAGTGTGTGGGCAGGATTG | 6531 | CTCTTTCTCGTTATTCAGATTG | 20+52 |
