# Supplementary material for: Genome Sequence of Saccharomyces carlsbergensis, the World’s First Pure Culture Lager Yeast
Source: G3 (Bethesda). 2014 Feb 27;4(5):783–93. doi: 10.1534/g3.113.010090 (PMC4025477; doi:10.1534/g3.113.010090)
Supplement: Supporting Information [file supp_g3.113.010090_TableS2.pdf]

Table S2 Volatile compound analysis

compound		WS34/70			CBS1513			CBS1503		
Ethanol	% v/v	5,86	±	0,01	6,04	±	0,01	5,13	±	0,00
acetaldehyde	ppm	3,5	±	0,7	5,2	±	0,2	4,5	±	0,2
ethylacetate	mg/l	35,1	±	1,3	18,1	±	1,3	17,5	±	4,4
isobutanol	mg/l	116,8	±	11,0	194,9	±	1,4	76,6	±	27,2
isobutylacetate	mg/l	0,3	±	0,1	0,2	±	0,1	0,1	±	0,1
propanol	mg/l	31,0	±	1,1	25,4	±	0,3	24,8	±	1,2
isoamyl alcohol	mg/l	244,1	±	17,2	224,3	±	1,2	174,0	±	32,1
isoamylacetate	mg/l	7,5	±	0,8	4,9	±	0,4	2,5	±	1,1
2-phenylethanol	mg/l	57,8	±	6,4	76,9	±	7,2	60,1	±	17,9
2-phenylethyl acetate	mg/l	1,3	±	0,1	1,3	±	0,1	0,7	±	0,3
ethyl hexanoate	mg/l	0,2	±	0,1	0,2	±	0,0	0,2	±	0,1
ethyl octanoate	mg/l	0,2	±	0,0	0,2	±	0,0	0,1	±	0,0
hexanoic acid	mg/l	0,8	±	0,0	0,7	±	0,1	0,7	±	0,2
octanoic acid	mg/l	5,4	±	0,2	6,5	±	0,3	5,6	±	1,6
vinyl gaïacol	mg/l	0,7	±	0,1	0,7	±	0,1	0,8	±	0,0
Decanoic acid	mg/l	0,2	±	0,0	1,3	±	0,1	1,3	±	0,3
Total acids	mg/l	6,4	±	0,2	8,5	±	0,3	7,6	±	1,6
Sub-total alcohols	mg/l	205,6	±	17,8	297,3	±	8,8	161,4	±	44,7
Total esters	mg/l	43,3	±	2,2	23,7	±	1,8	20,3	±	5,5
