# Supplementary material for: Genome Sequence of Saccharomyces carlsbergensis, the World’s First Pure Culture Lager Yeast
Source: G3 (Bethesda). 2014 Feb 27;4(5):783–93. doi: 10.1534/g3.113.010090 (PMC4025477; doi:10.1534/g3.113.010090)
Supplement: Supporting Information [file supp_g3.113.010090_TableS3.pdf]

Table S3 Scaffold assembly for *S. carlsbergensis*

Saccharomyces carlsbergensis			
Sc Chr	Scaffolds	Se Chr	Scaffolds
I	33	I	34
II	7	II-IV	1
III	38 (TY) 35	III	31+78+35part
IV	3+42+1part	IV-II	4
V	12	V	13
VI	-	VI	29
VII	6+32+77+59	VII	2
VIII	50+22**48	VIII-XV	27+15
IX	19	IX	21
X	24 (TY) 23	X	25*37+28
XI	-	XI	53*10, 52
XII	-	XII	5+40
XIII	39+51 (TY) 11+41+47	XIII	39+30 (TY) 16+47
XIV	46 (TY) 14 (TY) 36	XIV	8
XV	44+17+15	XV-XIII	9+43
XVI	56+18*20+52	XVI	56+18+26+45+52

A "+" indicates that scaffolds were combined by PCR and sequencing. Grey boxes mark scaffolds that represent either chromosomes consisting of only one scaffold or chromosomes which were generated by merging scaffolds. Red boxes mark scaffolds with gaps containing TY-elements. A "*" indicates a gap < 0.5 kb verified by PCR and "**" indicates the region between YHR165C and YHR174W that is apparently missing in *S. carlsbergensis*.
